# Computed Tomography and Magnetic Resonance Imaging Findings in a Case with Biliary Microhamartomas

**DOI:** 10.1155/2012/976078

**Published:** 2012-01-31

**Authors:** Alper Dilli, Umit Yasar Ayaz, Ilhami Yüksel, Cagrı Damar, Sevin Ayaz, Baki Hekimoglu

**Affiliations:** ^1^Department of Radiology, Ministry of Health, Diskapi Yildirim Beyazit Training and Research Hospital, Altindag, O6110 Ankara, Turkey; ^2^Department of Radiology, Ministry of Health, Mersin Women's and Children's Hospital, Halkkent, 33240 Mersin, Turkey; ^3^Department of Gastroenterology, Ministry of Health, Diskapi Yildirim Beyazit Training and Research Hospital, Altindag, O6110 Ankara, Turkey; ^4^Department of Nuclear Medicine, Mersin State Hospital, Ministry of Health, 33050 Mersin, Turkey

## Abstract

Biliary microhamartomas, also known as bile duct hamartomas and von Meyenburg complexes, are benign neoplasms containing cystic dilated bile ducts embedded in fibrous stroma. They develop in hepatobiliary system, do not generally give clinical outcomes, and are detected incidentally. However, they can rarely show malignant transformation. Our aim was to report the contribution of computed tomography, routine magnetic resonance imaging, and magnetic resonance cholangiopancreatography in the diagnosis of biliary microhamartomas in a 61-year-old woman.

## 1. Introduction

Biliary microhamartomas, also known as bile duct hamartomas and von Meyenburg complexes, develop in hepatobiliary system and do not generally give clinical outcomes. They are detected incidentally in most cases. Biliary microhamartomas are focal, disorderly collection of bile ducts that results from failure of involution of embryonic bile ducts [1]. They are benign neoplasms; however, they can rarely show malignant transformation [2]. Biliary microhamartomas are not uncommon. At autopsy, they were found in 5.6% of adults and in 0.9% of children [3]. Their incidence was reported as 0.6% in a serie including 2000 consecutive liver needle biopsies [4]. Our aim was to report the contribution of computed tomography (CT), routine magnetic resonance imaging (MRI), and magnetic resonance cholangiopancreatography (MRCP) in diagnosis of biliary microhamartomas.

## 2. Case Report

**Figure 1:**
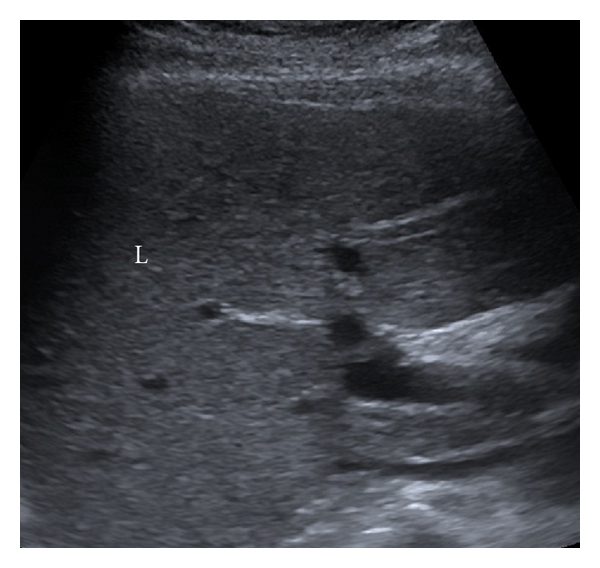
Ultrasonographic image of liver (L). No prominent lesion could be demonstrated.

A 61-year-old, mildly obese woman with short stature was referred with right upper quadrant pain. The history of the patient was unremarkable, and she did not mention any medical or surgical illness. Laboratory examinations revealed hemoglobin (Hb): 13.6 g/dL, white blood cell (WBC): 7,100/mm³, serum albumin: 3.7 g/dL (N: 3.8–5.1 g/dL), alanine aminotransferase (ALT): 24 U/L (N: 0–31 U/L), alkaline phosphatase: 202 U/L (N: 0–240 U/L), gamma-glutamyl transferase (GGT): 24 U/L (N: 0–38 U/L). The patient was informed about the imaging procedures, and consent was obtained from her. All the procedures were performed according to the World Medical Association Declaration of Helsinki. Initially, we performed abdominal ultrasonography (US), but we were unable to demonstrate any prominent lesion in liver (Figure 1). 

**Figure 2:**
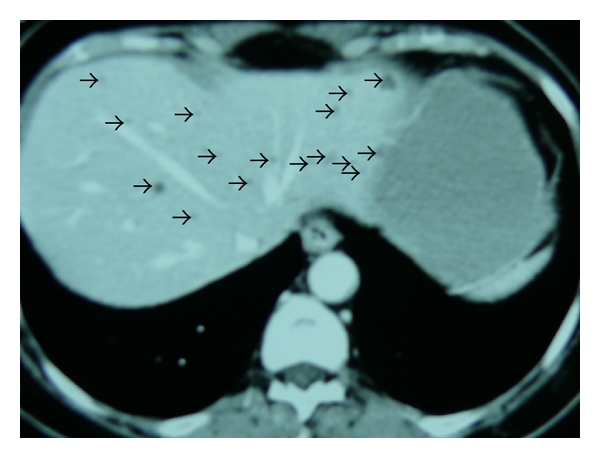
Contrast-enhanced CT revealed multiple, scattered, small, hypodense nodular lesions in liver (black arrows).

CT, routine MRI, and MRCP were performed afterwards. Contrast-enhanced CT revealed multiple, scattered, small, hypodense nodular lesions in liver parenchyma, measuring 1.5 cm or less in size (Figure 2). 

**Figure 3:**
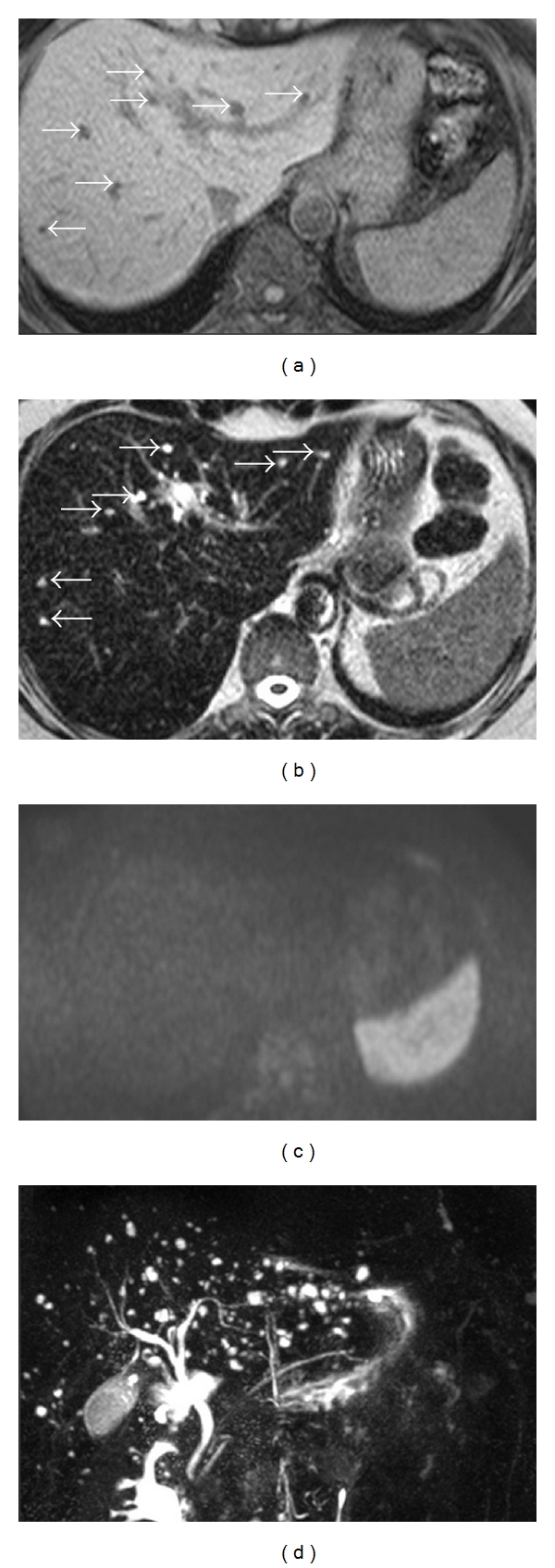
On T1-weighted image of the liver (a), multiple, small, hypointense nodular lesions with slightly irregular contours (white arrows) are demonstrated, which are hyperintense (b) (white arrows) on T2-weighted image. Diffusion-weighted image (c) reveals no restriction of diffusion in the liver lesions. MRCP (d) depicts an abundant number of hyperintense small lesions allover the liver which show no association with the biliary system. The widths and contours of intrahepatic and extrahepatic bile ducts and pancreatic canal are normal.

On upper abdominal MRI, multiple, small, nodular lesions with slightly irregular contours in the liver parenchyma, measuring 1.5 cm or less in size, were detected. The lesions were hypointense on T1-weighted images and hyperintense on T2-weighted images. Diffusion-weighted images revealed no restriction of diffusion in the liver lesions. MRCP depicted an abundant number of hyperintense small lesions allover the liver which showed no association with the biliary system. The widths and contours of intrahepatic and extrahepatic bile ducts and pancreatic canal were normal on MRCP (Figure 3). 

After making a detailed differential diagnosis, clinical and imaging findings were consistent with biliary microhamartomas, and the patient was included in a follow-up program to monitor the liver lesions.

## 3. Discussion

Biliary microhamartomas are tumor-like lesions of the liver [2]. Histologically, they are clusters of proliferated bile ducts lined by single layer of cuboidal cells embedded in fibrocollagenous tissue, and their size varies between 0.1 and 10 mm, but lesions measuring 1.5 cm in diameter can be demonstrated, as it was in the present case. Biliary microhamartomas do not generally give any symptoms [1, 5]. Adult polycystic liver disease (APLD) was reported to associate with biliary microhamartomas [1, 6]. With any imaging modality mentioned above, we could not be able to demonstrate larger and more numerous liver cysts which we are used to see in enlarged, diffusely cystic livers with APLD. But we decided to include the patient in a follow-up program to monitor the liver lesions.

US, CT, MRI, MRCP, and hepatobiliary scintigraphy (HBS) were used as imaging modalities in the diagnosis of biliary microhamartomas [7–9]. On US, the lesions can be seen as multiple, small, hypoechoic, or anechoic cystic lesions involving all segments. However, besides small cystic lesions, biliary microhamartomas can also be seen as hyperechogenic areas or lesions, and comet-tail artifacts may be seen posterior to the lesions [9, 10]. In the present case, US did not reveal any prominent lesion, suggesting that US may not be a suitable modality to show biliary microhamartomas. On CT, biliary microhamartomas are observed as multiple, round or irregular, small, hypodense lesions up to scattered throughout the liver on precontrast images. Luo et al. reported that on CT, lesions showed no enhancement, but increased in number by approximately 80–150% after administration of intravenous contrast medium [11]. On MRI, lesions appear hypointense and hyperintense on precontrast T1-weighted images and T2-weighted images, respectively. They show no communication with the biliary tree and demonstrate irregular delineation with no or slight rim enhancement following gadolinium administration [7].

In differential diagnosis, it is necessary to consider peribiliary cysts, simple hepatic cysts, autosomal dominant polycystic disease, metastatic liver disease, microabscesses, dilated biliary ducts, and Caroli's disease [1, 8, 9, 12]. Peribiliary cysts are multiple, small, cystic dilatations of the intrahepatic extramural peribiliary glands, but they are located exclusively in the hepatic hilum and along the larger portal tract [13], which is different from the scattered distribution of biliary microhamartomas. Simple hepatic cysts are not as numerous and uniformly small as biliary microhamartomas. In autosomal dominant polycystic disease, cysts are usually larger and more numerous. Liver metastases are more variable in size, density, or signal intensity on CT and MRI [1]. MRI can aid in the differentiation of liver cysts (such as biliary microhamartomas) and liver metastases by identifying the hyperintense signal from liver cysts on heavily T2-weighted sequences [8]. Differentiation of biliary microhamartomas from microabscesses can generally be made clinically. Differentiation from dilated biliary ducts and Caroli's disease can be made by looking at their association with biliary ducts using MRCP. Nuclear imaging can also be performed in differentiation of biliary microhamartomas from Caroli's disease. In Caroli's disease, HBS shows a particular pattern of cystic dilatation with accumulation of tracer in the intrahepatic ducts of the biliary tree [14]. Zheng et al. performed HBS in a case of biliary microhamartoma by using 99mTC-N-pyridoxy 1-5-methyl tryptophan (99mTC-PMT). HBS revealed that biliary system excreting function of radioisotope was normal and demonstrated normal appearances of biliary system without pooling areas [9] which helped to differentiate the entity from Caroli's disease. In our case, since MRCP depicted bile ducts with their normal width, shape, and contours, we did not need to perform further imaging with HBS. We recommend the clinicians to use MRCP as an ultimate imaging modality to differentiate biliary microhamartomas from Caroli's disease as we did in our case, but whenever they experience difficulty in diagnosis, we suggest that they can use HBS as a confirmative tool to solve the problem.

In conclusion, CT, MRI, and MRCP were useful in differentiating biliary microhamartomas from other cystic liver diseases and metastatic liver lesions in our case. Though biliary microhamartomas rarely show malignant transformation, this probability make radiological imaging of biliary microhamartomas necessary for early diagnosis.

##  Conflict of Interests

The authors declared that they have no conflict of interests.

##  Disclosure

This case report was presented in 16th Annual Meeting of Turkish Society of Magnetic Resonance, 19–21 May 2011, Istanbul.
